# Development of the celiac disease symptom diary version 2.1^©^ (CDSD 2.1^©^) patient-reported outcome measure

**DOI:** 10.1007/s11136-024-03799-6

**Published:** 2024-10-26

**Authors:** Kellee Howard, Daniel Adelman, Sonal Ghura, Sarah Acaster, Sarah Clifford, Ciaran P. Kelly, Susan A. Martin, Lisa M. Meckley, Daniel A. Leffler

**Affiliations:** 1 ICON plc, San Fransisco, CA USA; 2 Present Address: Patient Centered Solutions, IQVIA, Montreal, QC Canada; 3 University of California, San Fransisco, CA USA; 4 Takeda Development Center Americas Inc, Cambridge, CA USA; 5 Oxford Outcomes Ltd. (later acquired by ICON plc), Oxford, UK; 6 Present Address: Acaster Lloyd Consulting Ltd., London, UK; 7 Present Address: Sprout Health Solutions, Los Angeles, CA USA; 8 Celiac Center, Beth Israel Deaconess Medical Center, Harvard Medical School Celiac Research Program, Boston, MA USA; 9 RTI Health Solutions, Ann Arbor, USA MI; 10 Takeda Development Center Americas Inc., Cambridge, MA USA

**Keywords:** Celiac disease, Coeliac disease, CDSD, Patient-reported outcome, Symptoms

## Abstract

**Purpose:**

For patients with celiac disease (CeD), the only current management option is adherence to a strict gluten-free diet (GFD); however, many patients on a GFD continue to experience symptoms with a significant impact on quality of life. Potential new treatments for CeD are under development and a validated patient-reported outcome measure is required to evaluate their utility in clinical trials. The purpose of this article is to provide a history of the development of the Celiac Disease Symptom Diary (CDSD) 2.1^©^ for use in clinical trials.

**Methods:**

Qualitative and quantitative studies were conducted from 2010 to 2021, including concept elicitation and cognitive debriefing interviews with adult and adolescent participants with CeD (*N* = 93) diagnosed via biopsy and/or serology and input from eight interviews with CeD clinical experts. During these studies, different iterations of the CDSD were presented to the US Food and Drug Administration and the European Medicines Agency, and modifications were made in line with their feedback.

**Results:**

These studies ultimately led to the development of CDSD 2.1^©^, a daily diary which focuses on key symptoms of CeD (abdominal pain, bloating, diarrhea, nausea and tiredness). This patient-reported outcome measure was readily understood by adult and adolescent participants with CeD and content validity was demonstrated in both populations.

**Conclusion:**

CDSD 2.1^©^ is a content-valid patient-reported outcome measure developed in accordance with best practices and regulatory guidance. A thorough exploration of the psychometric properties of CDSD 2.1^©^ for both adult and adolescent participants with CeD is ongoing to support utilization in clinical trials.

**Supplementary Information:**

The online version contains supplementary material available at 10.1007/s11136-024-03799-6.

## Introduction

Celiac disease (CeD) is a chronic immune-mediated disorder triggered by the ingestion of gluten in genetically predisposed individuals. CeD has a global prevalence of approximately 1% with reported regional prevalence ranging from 0.5% to 1.4% in the USA and 0.7% to 1.3% in Europe [1–3].

CeD is characterized by damage to the small intestine and symptoms can include abdominal pain, diarrhea, constipation, vomiting/nausea, bloating/flatulence, fatigue, headaches and joint pain [1, 4]. Complications of CeD are diverse and include malabsorption and malnutrition, anemia, osteoporosis, neurological symptoms, dermatitis herpetiformis, infertility and certain hematological malignancies [1, 4].

The only current management option available for CeD is adherence to a lifelong strict gluten-free diet (GFD); however, many patients continue to experience symptoms and/or intestinal injury owing to inadvertent gluten exposure as well as having a substantial burden imposed by adhering to a strict GFD [2, 4, 5]. CeD symptoms have been shown to have a significant effect on patients’ health-related quality of life (HRQoL) [6, 7]. Studies conducted with adults and adolescents to develop a conceptual model of the impact of CeD revealed the negative impact that CeD symptoms can have on physical functioning, sleep, daily activities, social activities, emotional functioning and relationships [5]. For those patients who respond well to a GFD, despite a reduction in symptoms, many report a persistent impact of CeD on their HRQoL owing to anxiety around inadvertent gluten exposure and lifestyle impacts of adhering to a GFD [5, 8].

To address the unmet need for effective therapies for patients with CeD on a GFD, several treatment options are under development and validated patient-reported outcome (PRO) measures are required to evaluate their efficacy [9]. To comply with the stringent guidelines from regulators on methods for the evaluation of treatment benefits and effectiveness, a fit-for-purpose CeD PRO measure must demonstrate content validity, construct validity and an ability to detect change in relevant symptoms of CeD in the target population [10–12].

Several PRO measures have previously been used to measure the benefit of potential CeD treatments: Celiac disease-specific modification of the Gastrointestinal Symptoms Rating Scale (GSRS); Psychological General Well-Being (PGWB) Index; Celiac Disease Patient Reported Outcome (CeD PRO); Coeliac Disease Quality of Life (CD QoL) survey; Coeliac Disease Assessment Questionnaire (CDAQ); and Celiac Symptom Index (CSI) [13, 14]. Although some of these PRO measures contain items that evaluate important concepts in CeD, the majority have not been fully developed to meet the stated expectations of the US Food and Drug Administration (FDA) for regulatory purposes [14, 15].

Symptom-related PRO measures are generally accepted as appropriate primary or co-primary endpoints in CeD clinical studies because active CeD is often associated with a high symptom burden [16, 17]. In a draft guidance document from the FDA on the development of drugs for CeD as adjunctive treatment to a GFD (published in April 2022), it is recommended that clinical outcome assessments in CeD clinical trials include a PRO instrument that measures the core signs and symptoms of CeD with daily assessments (24-h recall period) as co-primary endpoints with histology measures, and HRQoL impacts as secondary endpoints [18].

FDA guidelines describe the iterative process that should be followed during PRO instrument development [11]. A preliminary PRO instrument should be developed on the basis of a conceptual framework of the disease (derived from literature and expert reviews and modified as required after patient input) and tested in a relevant patient population to assess content validity and confirm understanding of instructions and appropriate responses to the questions. In response to feedback from qualitative patient interviews and regulatory agencies, the instrument should be modified as required (e.g. changes to items, wording, response options, scoring and recall period). The modified instrument should then be re-assessed in further qualitative patient interviews and this process repeated until the instrument is deemed fit-for-purpose by all stakeholders.

This article describes the historical development and iterative modification of the Celiac Disease Symptom Diary (CDSD), a daily diary that focuses on the key symptoms of CeD including abdominal pain, bloating, diarrhea, nausea and tiredness. During development, different iterations of the CDSD were presented to the FDA and the European Medicines Agency (EMA); modifications to the measure were made in line with their feedback, with the ultimate aim of using the CDSD as a primary endpoint in registrational trials of new CeD therapies.

## Methods

**Fig. 1:**
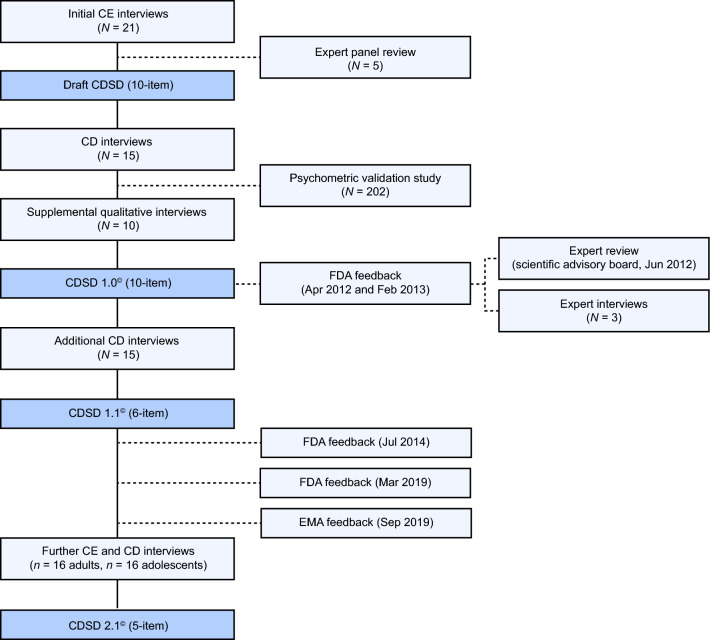
CDSD development stages. *CD* cognitive debriefing, *CDSD* celiac disease symptom diary, *CE* concept elicitation, *EMA*, European medicines agency, *FDA* food and drug administration

Symptom items included in the CDSD and subsequent iterative drafts were informed by direct input from patients with CeD (*N* = 61), eight interviews with CeD clinical experts and feedback from the FDA. This work led to the development of the earlier versions of the CDSD (CDSD 1.0^©^ and CDSD 1.1^©^). In response to additional FDA and EMA feedback, further patient interviews (*N* = 32) and refinements to the CDSD were carried out to generate CDSD 2.1^©^ (Fig. 1).

The qualitative and quantitative studies conducted from 2010 to 2021 are detailed in Abstracts 1–3 in the Online Resource. The content of these abstracts is referenced as we describe the various versions of the CDSD and how they affected the evolution of the CDSD to its current version (CDSD 2.1^©^). The CDSD development and evaluation process was guided by the FDA, EMA and International Society for Pharmacoeconomics and Outcomes Research (ISPOR) recommendations for developing PRO measures [17, 19, 20].

## Results

### CDSD 1.0 (Abstract 1, online resource)

#### Qualitative studies for content validation

Development of the CDSD was initiated in 2010 with concept elicitation interviews conducted with participants with biopsy-confirmed CeD (*N* = 21) and input from clinical experts (*N* = 5; four US gastroenterologists and one US registered dietitian specializing in CeD); Fig. 1. Concept saturation for the common and less-frequently reported symptom groups was reached by the sixth and twentieth interviews, respectively; see **Online Resource Abstract 1** for full details. Eleven symptoms were spontaneously reported by participants with CeD: abdominal pain, bloating, cognitive difficulties, constipation, diarrhea, fatigue/tiredness, headache, nausea, passing gas, joint pain and skin rash. Clinical experts subsequently endorsed 10 out of 11 of these symptoms but recommended that joint pain be removed because it was not considered to be reliably attributable to CeD and gluten exposure. This resulted in a draft 10-item PRO measure that measured symptom severity using a scale of 0–10 of ‘no pain’ to ‘worst imaginable pain’ for abdominal pain and headache, or a 5-point Likert scale of ‘very mild’ to ‘very severe’ for boating, constipation, diarrhea, nausea, skin rash and tiredness. A 5-point scale of ‘not at all’ through to ‘completely’ was used to measure cognitive difficulties/difficulty thinking clearly and impact of symptoms on activities and sleep. The frequency of diarrhea (with descriptive options for stool consistency) and spontaneous bowel movements was measured using a numerical scale. This preliminary CDSD was designed to be administered via an interactive voice recognition system (IVRS) with a 24-h recall period.

The preliminary CDSD was subsequently reviewed in cognitive debriefing interviews in participants with CeD (*N* = 15) to confirm concept relevance and to assess any ambiguities in meaning and interpretation. These interviews identified some areas of ambiguity within the diarrhea and constipation items. Although the term ‘diarrhea’ was understood with the descriptor ‘loose stool,’ there was uncertainty around the conceptual linkage with severity. Likewise, ‘constipation’ was understood by all participants, but the term ‘successful bowel movement’ in the follow-up question was not understood. Therefore, 10 supplemental qualitative interviews were conducted in which participants’ understanding of diarrhea severity and interpretation and relevance of the constipation item were specifically explored. An initial psychometric study of the CDSD was also conducted in participants with biopsy-confirmed CeD (*N* = 202) recruited at three US clinical sites (Beth Israel Deaconess Medical Centre, Boston; Mayo Clinic, Minnesota; or Columbia University, New York). Passing gas, abdominal pain, fatigue and bloating were the most commonly endorsed symptoms across all 7 days of the psychometric study. This study also indicated a poor sensitivity in discriminating between different levels of symptom severity related to constipation/unsuccessful bowel movements, abdominal pain and diarrhea.

The main change introduced in response to the supplemental qualitative interviews was the replacement of diarrhea and constipation sub-items (i.e. loosest stool consistency and number of successful bowel movements) with general severity rating items. Follow-up questions on the impact of symptoms on sleep were also removed. These changes resulted in a 10-item tool, CDSD 1.0^©^.

Demographics and clinical characteristics of interview participants, frequency of spontaneously elicited symptoms and symptom saturation matrices for the initial and supplemental interviews and results from the psychometric study, conducted during development of CDSD 1.0^©^ are provided in the **Online Resource** (**Abstract 1)** along with a sample copy of this earlier version of the CDSD (for illustrative purposes only).

#### FDA feedback on CDSD 1.0 and corresponding changes

FDA feedback (April 2012) on CDSD 1.0^©^ and expert input (scientific advisory board and expert interviews with two US gastroenterologists and one US registered dietitian specializing in CeD, June 2012) on the supplemental interview findings and the psychometric study highlighted the need to conduct further interviews.

At this stage, the FDA did not evaluate content validity of the draft measure and stated that an adequately established and portrayed conceptual framework for CeD would be required to confirm content validity. Studies to support the development of a conceptual model for CeD were undertaken and subsequently reported by Leffler et al. in 2017 [5].

Further recommendations from the FDA on CDSD 1.0^©^ (February 2013) included removing symptom impact sub-questions and focusing on the presence/severity of gastrointestinal (GI) symptoms typically experienced by patients (i.e. eliminating concepts not deemed to be directly associated with the GI effects of CeD and/or unlikely to improve in a clinical study). In response, cognitive difficulties, headache, passing gas and skin rash were removed and all impact and duration sub-items were removed.

The FDA also recommended replacing the word ‘fatigue’ with ‘tiredness’ and suggested that the wording related to spontaneous bowel movements, diarrhea and bloating be refined and/or definitions be included to clarify their meaning (pending further qualitative studies). In response, further clarifications were made to the wording of the CDSD. The questionnaire was also modified such that the constipation item was skipped if a patient answered ‘yes’ to experiencing diarrhea or spontaneous complete bowel movements.

The FDA also recommended that reference to CeD be removed when asking patients about a specific symptom, because patients should be asked to report on a symptom without having to make an assessment concerning its cause or origin. This change was also accepted and implemented.

For some of the response options it was unclear to the FDA whether or not patients could reliably distinguish between ‘very mild’ and ‘mild’ or between ‘very severe’ and ‘severe.’ The FDA also deemed that the scoring algorithm was inadequate because response options were transformed to a 0–10 scale that resulted in assigning a score of zero for very mild or infrequent symptoms (e.g. having diarrhea once or twice). The FDA suggested that a score of zero should be used to indicate the absence of a symptom and further assessment of the response options was recommended by the FDA to implement the necessary refinements. The FDA also indicated that the original number of questions included would be too burdensome for a daily diary and reiterated that removal of non-GI symptom items would help to reduce respondent burden.

### CDSD 1.1^©^ (Abstract 2, online resource)

#### Qualitative studies for content validation

After modifications of the CDSD in response to the above FDA feedback, the resulting PRO measure had 6 items with response options that included a numerical scale (0–10) for abdominal pain, a 5-point Likert scale (very mild to very severe) for bloating, nausea and tiredness, and frequency options (once to ≥ 10 times) for diarrhea and spontaneous bowel movement.

These modifications were assessed in additional cognitive debriefing interviews in adult participants with CeD (*N* = 15). These interviews confirmed that the content of the revised PRO measure (CDSD 1.1^©^) was clear and comprehensible, focused on symptom concepts central to the typical participant’s experience of CeD and that this PRO measure could be appropriately interpreted and administered. Demographics and clinical characteristics of participants in these cognitive debriefing interviews are provided in **Online Resource Abstract 2**, along with a sample copy of CDSD 1.1^©^ (for illustrative purposes only).

#### FDA feedback on CDSD 1.1^©^

FDA feedback on CDSD 1.1^©^ (July 2014) suggested that additional validation evidence was necessary to use the CDSD to support product labeling of potential CeD treatments for adults and adolescents. FDA recommendations after a further review of CDSD 1.1^©^ (March 2019) included an assessment of this PRO measure in younger adolescents (aged 12–14 years) to confirm content validity and understanding in this age group.

The FDA also suggested that the severity scales and frequency options should not be combined into a single score (in particular, that diarrhea frequency and non-stool GI severity items should be analyzed separately). The FDA again requested that evidence from participants’ interviews be provided to confirm that patients with CeD can distinguish between the response options of ‘very mild’ and ‘mild’ and between ‘very severe’ and ‘severe,’ and a reduction in response options should be considered accordingly. It was also suggested that nausea and bloating severity scores should include zero (for patients who report none of these symptoms) and abdominal pain scoring should align with nausea and bloating scores (0 to 5).

In response to a direct question, the FDA agreed that the Bristol Stool Form Scale (BSFS) [21] could be used to ensure participants’ responses to the diarrhea question align with the relevant stool descriptors.

In addition, the FDA reviewed a Weekly CDSD Severity Score algorithm in which the daily symptom scores for all items were averaged across a 7-day period (proposed for use in clinical trials) and recommended that ‘tiredness’ should not be included in this weekly severity score (to focus potential clinical trial endpoints on GI symptoms only).

#### EMA feedback

The EMA’s Committee for Medicinal Products for Human Use (CHMP) provided feedback on CDSD 1.1^©^ in September 2019 and concluded that the disease concepts covered in this PRO measure may serve as a primary endpoint in CeD clinical studies. However, the CHMP pointed out that the impact of a GFD on patients’ lifestyle should also be considered in a CeD clinical trial.

The CHMP were asked whether the use of the BSFS would be suitable to ensure that patients’ response to the diarrhea question in CDSD 1.1^©^ aligns with the relevant stool descriptors in the BSFS. The CHMP agreed that the BSFS could be used in the context of daily evaluation as this scale was readily understood by both adult and adolescent populations (as demonstrated in cognitive debriefing interviews outside of the studies reported here). However, the use of the BSFS in addition to the diarrhea question (which already included a qualifier question on ‘loose, mushy or liquid’ bowel movements) was not readily understood.

The scoring and weighting across items included in CDSD 1.1^©^ were identified by the CHMP as an area that required further supportive evidence (e.g. the CHMP questioned the decision to cap the diarrhea count at nine episodes and suggested that all counts above nine should not be treated equally without evidence to support this decision).

Finally, the CHMP concluded that more studies may be required to confirm the adequacy of CDSD 1.1^©^ content for adolescent patients through further concept elicitation interviews with a larger number of participants from this younger population. The CHMP did not identify any issues with regard to patients’ comprehension of CDSD 1.1^©^.

### CDSD 2.1^©^ (Abstract 3, online resource)

#### Qualitative studies for content validation

In response to the FDA feedback regarding combining frequency and severity items, CDSD 1.1^©^ was further modified to focus on symptom severity scores only. Frequency of bowel movements was removed and a supplementary questionnaire (CDSD 2.1^©^—Frequency Supplement) was developed to capture information on the frequency of ‘all’ bowel movements and bowel movements classified as ‘Type 6 or 7’ on the BSFS; the use of the BSFS to assess the relationship between diarrhea severity and visual stool consistency was supported by the FDA and the EMA (along with removal of the ‘loose, mushy or liquid’ descriptors). Frequency of vomiting was also added to the CDSD 2.1^©^—Frequency Supplement. Furthermore, the severity scoring system for all items was modified to 0 (none), 1 (very mild), 2 (mild), 3 (moderate), 4 (severe) and 5 (very severe), and further testing was conducted to assess the relevance of the ‘very mild’ and ‘very severe’ response options. The non-GI item ‘tiredness’ was retained because this was reported with a higher frequency than other non-GI items and was considered of high importance to patients; however, it was not included in the Weekly CDSD Severity Score.

After the above changes, concept elicitation and cognitive debriefing interviews were conducted with adult (*N* = 16) and adolescent (*N* = 16) participants with CeD. Targeted recruitment of younger adolescent participants (to ensure comprehension of items in this age group) was successful, with half of adolescent participants aged 12–14 years (*n* = 8). The inclusion criteria used were aimed at reflecting patient populations in planned CeD clinical trials (see **Online Resource Abstract 3**).

During an initial concept elicitation portion of the interviews, participants were asked to report on the symptoms they experienced as well as identifying symptoms that were most bothersome to them. A cognitive debriefing portion of the interview was then initiated in which participants were asked to review the modified CDSD in its entirety for content validity, along with the CDSD 2.1^©^—Frequency Supplement. Refinements to the CDSD were made between each of three rounds of interviews as required. Details regarding participant feedback from the concept elicitation and cognitive debriefing interview rounds are provided in **Online Resource Abstract 3** along with a sample copy of CDSD 2.1^©^ and the CDSD 2.1^©^—Frequency Supplement (for illustrative purposes only and not to be used without permission from the CDSD’s licensor and distributor, Mapi Research Trust [https://eprovide.mapi-trust.org/]).

Modifications based on these interviews included a minor change to further specify ‘each evening’ in the instructions for completing the questionnaire. Constipation was removed as a severity assessment because the question on bowel movement frequency included in the CDSD 2.1^©^—Frequency Supplement was felt to address this concept better. The response option ‘very mild’ was also removed for all items (as previously recommended in FDA feedback and confirmed as a suitable omission in these interview rounds in which several participants reported an inability to distinguish between ‘very mild’ and ‘mild’). Finally, in response to adolescent feedback, the word ‘belly’ was added in parentheses after the term ‘abdominal,’ for clarification. These final interviews demonstrated that CDSD 2.1^©^ was readily understood by both adult and adolescent participants.

A Weekly CDSD Severity Score has been proposed for use in clinical trials in which the daily symptoms scores for all GI items are averaged across a 7-day period. As per FDA recommendations, the Weekly CDSD Severity Score does not include the ‘tiredness’ item.

## Discussion

The goal of this article is to present a consolidated historical description of the development of a novel PRO measure (CDSD 2.1^©^) to evaluate the outcomes of new therapies for CeD in a clinical trial setting. The 2022 FDA draft guidance ‘Celiac Disease: Developing Drugs for Adjunctive therapy to a Gluten-Free Diet’ recommends the use of a well-defined and reliable PRO instrument to measure the core signs and symptoms of CeD with a 24-h recall period as a co-primary endpoint in clinical trials and CDSD 2.1^©^ was developed to addresses this requirement [18].

In CeD, both symptoms and maintaining a GFD have a substantial impact on patient HRQoL and it is therefore acknowledged that HRQoL should also be assessed in CeD clinical studies to understand the impact of interventions on the lives of patients with this complex disease [5]. This is supported in the FDA’s latest draft guidance in which a score to measure patient HRQoL (specifically impacts of CeD signs or symptoms on patients’ daily lives) is suggested as a secondary endpoint in CeD clinical trials [18]. The EMA also noted (as part of their feedback on the CDSD 1.1^©^) that the impact of a GFD on patients’ lifestyle should be considered in CeD clinical trials.

CDSD 2.1^©^ measures the severity of the core GI symptoms of CeD (diarrhea, abdominal pain, bloating and nausea) and tiredness. These GI symptoms have been shown to affect sleep (e.g. disrupted sleep owing to abdominal pain), daily or social activities (e.g. experiencing diarrhea or nausea can lead to patients needing to stay near a bathroom and not being able to engage in certain activities and abdominal pain can prohibit patients from being able to carry out usual daily activities), and emotional functioning (e.g. depression and anxiety around diarrhea and abdominal pain) [5]. Although FDA feedback during development of the CDSD recommended a focus on GI symptoms, tiredness is also included in the CDSD because, during the qualitative studies described here, this symptom was reported with a higher frequency or was considered of higher importance by participants than other non-GI symptoms. Tiredness has also been shown to be one of the most commonly reported symptoms in studies to develop a conceptual model of CeD [5, 8]. Tiredness was shown to affect patients’ physical functioning, in particular limiting their participation in sports or exercise and social activities, hence this is considered an important symptom to measure [5].

In addition, three frequency items are included in a supplementary questionnaire (CDSD 2.1^©^—Frequency Supplement): vomiting, bowel movements (all), and bowel movements classified as Type 6 or 7 on the BSFS. These frequency measures are separate from the severity measures in the main CDSD as recommended by the FDA.

CDSD 2.1^©^ was developed in accordance with the FDA’s 2009 ‘Guidance for Industry on Patient-Reported Outcome Measures: Use in Medical Product Development to Support Labeling Claims’ [11] and aligns with subsequent draft guidance documents on patient-focused drug development: ‘Methods to Identify What is Important to Patients’ [22] and ‘Selecting, Developing, or Modifying Fit-For-Purpose Clinical Outcome Assessments’ [10] (both published in 2022). After refinements based on direct FDA and EMA feedback, CDSD 2.1^©^ was readily understood by adult participants and adolescent participants as young as 12 years, and content validity has been demonstrated in both populations.

CDSD 2.1^©^ is the latest version of this PRO and is the version that should be used henceforth. CDSD 2.1^©^ is available via the non-profit Mapi Research Trust (Lyon, France; https://eprovide.mapi-trust.org/instruments/celiac-disease-symptom-diary-version-2.1-c) for which a license for use in approved research studies can be sought (see more information in section ‘Copyright and conditions of use of the CDSD and its forms/derivatives’ at the end of this article). The status of available translations can be checked with the Mapi Research Trust. CDSD 2.1^©^ has been designed for used in electronic platforms (rather than an IVRS) to facilitate future use in clinical trials, which follows industry guidelines and standard practice [23].

Limitations of these studies include the lack of ethnic diversity among the enrolled participants who were predominantly non-Hispanic White. However, the ratio of non-Hispanic White to Hispanic participants in these studies is believed to be representative of patients with CeD typically seen in the clinic [24]. Furthermore, the adult population in these studies were predominantly college or university educated which may introduce bias. The FDA’s advice to restrict the scope of CDSD 2.1^©^ to GI symptoms and reduce the respondent burden was intended to optimise the reliability of the instrument, however this limits the domains that the PRO measure can assess. Psychometric validation of CDSD 2.1^©^ is required to confirm that the PRO measure can detect changes in CeD symptom severity in response to treatment. This is currently being evaluated in a 12-week observational study of patients with symptomatic CeD (NCT05309330) [25] and in a phase 2 trial of a potential therapy for CeD (NCT05353985).

## Conclusions

CDSD 2.1^©^ was designed to evaluate symptoms of CeD in the context of clinical trials in adults and adolescents aged from 12 years. It was developed in accordance with best practices and subsequently refined in response to feedback from the FDA and EMA. CDSD 2.1^©^ is now undergoing further psychometric evaluation to assess measurement properties in CeD clinical trials that are determining treatment effectiveness.

## Supplementary Information

Below is the link to the electronic supplementary material.

Supplementary file1 (DOCX 766 KB)

## Data Availability

The data that support the findings of this study are available in the Online Resource for this article.
