# Supplementary material for: Development of the celiac disease symptom diary version 2.1© (CDSD 2.1©) patient-reported outcome measure
Source: Qual Life Res. 2024 Oct 26;33(12):3275–82. doi: 10.1007/s11136-024-03799-6 (PMC11599373; doi:10.1007/s11136-024-03799-6)
Supplement: Supplementary file 1 — Supplementary file1 (DOCX 766 KB) [file 11136_2024_3799_MOESM1_ESM.docx]

Online Resource

Supplement to: Development of the Celiac Disease Symptom Diary Version 2.1^©^ (CDSD 2.1^©^) patient-reported outcome measure

Submitted to: Quality of Life Research

Kellee Howard, Daniel Adelman, Sonal Ghura, Sarah Acaster, Sarah Clifford, Ciaran P. Kelly, Susan A. Martin, Lisa M. Meckley & Daniel A. Leffler

Corresponding author: Daniel A. Leffler, Takeda Development Center Americas Inc., 95 Hayden Avenue, Lexington, MA 02421, USA. Tel: +1 617 679 7323. Email: [daniel.leffler@takeda.com](mailto:daniel.leffler@takeda.com).

Contents:

Abstract 1: Development and validation of Celiac Disease Symptom Diary (CDSD) 1.0^©^

Abstract 2: Development and validation of CDSD 1.1^©^

Abstract 3: Development and validation of CDSD 2.1^©^

# Abstract 1: Development and validation of Celiac Disease Symptom Diary (CDSD) 1.0^©^

Objective:

To develop a patient-reported outcome (PRO) measure for symptoms of celiac disease (CeD).

Methods:

Development of the Celiac Disease Symptom Diary (CDSD) was initiated in 2010 with concept elicitation interviews conducted in participants with biopsy-confirmed CeD (*N* = 21; **Supplementary Table 1**) and input from clinical experts (*N* = 5; four US gastroenterologists and one US-registered dietitian specializing in celiac disease). Participants included in this study were aged 18 years or older, had biopsy-confirmed CeD and either positive serology for CeD or a response to a gluten-free diet (GFD) and were experiencing or had recently experienced (within the past 3 months) CeD-related symptoms. Participants were excluded if they were diagnosed with refractory CeD, irritable bowel syndrome or gastro-oesophageal reflux disease not explained by CeD, eosinophilic oesophagitis, microscopic colitis, Crohn’s disease or ulcerative colitis.

Cognitive debriefing interviews with a draft CDSD (to confirm concept relevance and to assess any ambiguities in meaning and interpretation) were subsequently conducted in participants with CeD (*N* = 15) applying the same inclusion/exclusion criteria as for the concept elicitation interviews.

An initial psychometric validation study of the draft CDSD developed from the above studies was conducted in participants with CeD (*N* = 202) recruited at three clinical sites in the USA (Beth Israel Deaconess Medical Centre, Boston; Mayo Clinic, Minnesota; and Columbia University, New York). Inclusion/exclusion criteria were the same as for the concept elicitation interviews. Participants completed the draft CDSD for 7 consecutive days.

A set of supplemental qualitative interviews was conducted in participants with CeD (*N* = 10) with the same inclusion/exclusion criteria employed as for the above studies. During these interviews, the interpretation and relevance of the constipation item and participants’ understanding of diarrhoea severity were specifically explored.

Results:

During the concept elicitation interviews, 11 common symptoms were spontaneously reported as well as impact of these symptoms on sleep (sleep disturbance); **Supplementary Table 2**. Concept saturation for the common and less-frequently reported symptom groups was reached by sixth and twentieth interviews, respectively (**Supplementary Table 2**).

A panel of five clinical experts reviewed the pool of the 11 spontaneously elicited common symptom concepts and recommended that 10 symptoms should be included. Joint pain was removed as it was not considered to be reliably attributable to CeD and gluten exposure. None of the 15 less-frequently mentioned ‘other’ symptoms were considered suitable for inclusion as they could not be attributed reliably to CeD.

The resulting draft CDSD included diarrhoea, abdominal pain, fatigue, bloating, constipation, headache, passing gas, skin rash, cognitive difficulties/difficulty thinking clearly and nausea, with follow-up questions on symptom severity, frequency and impact on activities and sleep. Response options included a scale of 0–10 for abdominal pain and headache (0 = no pain, 10 = worst pain participant could imagine), a 5-point Likert scale (very mild to very severe) for bloating, constipation, diarrhoea, nausea, skin rash and fatigue, a 5-point Likert scale (not at all to completely) for cognitive difficulties/difficulty thinking clearly and for impact of symptoms on activities and sleep, and a numerical response for frequency of diarrhoea (plus descriptive options for stool consistency) and spontaneous bowel movements. The preliminary CDSD was designed to be administered through an interactive voice response system (IVRS) with a recall period of 24 hours. In the subsequent cognitive debriefing interviews with 15 different participants (**Supplementary Table 3**), several areas of ambiguity were identified. The term ‘diarrhoea’ was understood by participants with the descriptor ‘loose stool’; however, there was uncertainty around the conceptual linkage with severity. The term ‘constipation’ was also understood, but ‘successful bowel movement’ in the follow-up question was not.

During the psychometric validation study (*N* = 202; **Supplementary Table 4**), passing gas, abdominal pain, fatigue and bloating were the most commonly endorsed symptoms across all 7 days of the study (**Supplementary Table 5** and **Supplementary Figure 1**). The least frequently reported symptoms were skin rash and headache, but there was no indication that any of these symptoms should be excluded owing to very low endorsement. Very low mean scores for sleep impact indicated a lack of relevance for this measure (follow-up question). This study also indicated a poor sensitivity in discriminating between different levels of symptom severity related to constipation/unsuccessful bowel movements, abdominal pain and diarrhoea. Supplemental qualitative interviews (*N* = 10; **Supplementary Table 6**) to further explore content validity did not reveal any new symptom concepts (**Supplementary Table 7**). In response to participants’ understanding of diarrhoea severity and interpretation and relevance of the constipation item, the main change introduced at this stage was the replacement of diarrhoea and constipation sub-items that were not very well understood (i.e. loosest stool consistency and number of successful bowel movements) with general severity rating items. The follow-up questions on the impact of symptoms on sleep were also removed. The resulting questionnaire was the CDSD 1.0^©^.

**Supplementary table 1** Development of the original 10-item CDSD (version 1.0)^©^: demographics and clinical characteristics for the concept elicitation interview population (*N* = 21)

| Characteristic | Concept elicitation interviews  (*N* = 21) |
| --- | --- |
| Age, years, mean (range) | 42 (19–85) |
| Female, % | 71 |
| Race/ethnicity, *n* (%) |  |
| White/non-Hispanic | 19 (90.5) |
| Asian | 1 (4.8) |
| Caribbean | 1 (4.8) |
| Education, *n* (%) |  |
| High school/GED | 3 (14.3) |
| Some college | 2 (9.5) |
| Trade school | 1 (4.8) |
| Associate degree | 1 (4.8) |
| Bachelor’s degree | 5 (23.8) |
| Master’s degree | 6 (28.6) |
| Professional/doctorate degree | 3 (14.3) |
| Employment status, *n* (%) |  |
| Employed full-time | 12 (57.1) |
| Employed part-time | 3 (14.3) |
| Student | 2 (9.5) |
| Retired | 2 (9.5) |
| Unemployed/seeking work | 1 (4.8) |
| Homemaker | 1 (4.8) |
| Physician reviewed biopsy indicating celiac disease | 21 (100) |
| GFD adherence, *n* (%) |  |
| All of the time | 19 (90.5) |
| Most of the time | 2 (9.5) |
| Comorbidities, *n* (%)^a^ |  |
| None | 6 (28.6) |
| Symptoms of GORD | 5 (23.8) |
| Symptoms of IBS | 6 (28.6) |
| Lactose intolerance | 7 (33.3) |
| Clinician’s Global Assessment of Severity,^b^ score, mean (range) | 4.1 (2–7) |
| Original presentation of symptoms, *n* (%) | |
| Classic celiac symptoms | 18 (85.7) |
| GI and neurological | 1 (4.8) |
| Leg/arm pains + GI | 1 (4.8) |
| Celiac crisis brought on by pregnancy | 1 (4.8) |

^a^Participants may report more than one comorbidity.

^b^Clinician’s Global Assessment of Severity was scored 1–7, where a higher score indicates a greater CeD severity.

*CDSD*, Celiac Disease Symptom Diary; *CeD*, celiac disease; *GED*, general equivalency diploma; *GFD*, gluten-free diet; *GI*, gastrointestinal; *GORD*, gastro-oesophageal reflux disease; *IBS*, inflammatory bowel syndrome.

**Supplementary table 2** Development of the original 10-item CDSD (version 1.0)^©^: frequency of spontaneously elicited symptom concepts and symptom saturation matrix from initial concept elicitation interviews (*N*= 21)

| Participant | 1 | 2 | 3 | 4 | 5 | 6 | 7 | 8 | 9 | 10 | 11 | 12 | 13 | 14 | 15 | 16 | 17 | 18 | 19 | 20 | 21 | Total (%) |
| --- | --- | --- | --- | --- | --- | --- | --- | --- | --- | --- | --- | --- | --- | --- | --- | --- | --- | --- | --- | --- | --- | --- |
| Diarrhoea | * | X |  | X | X | X | X | X | X |  | X | X | X |  | X | X | X | X | X |  | X | 17 (81) |
| Abdominal pain | * | X |  | X | X | X |  | X | X |  | X |  | X |  | X | X | X | X | X | X | X | 16 (76) |
| Fatigue |  | * | X | X |  | X | X |  |  | X | X |  | X | X | X |  | X | X |  |  |  | 12 (57) |
| Bloating | * | X | X |  |  |  |  |  |  |  | X | X |  |  | X |  | X | X | X | X | X | 11 (52) |
| Headache | * |  | X | X | X |  |  |  |  |  |  |  | X | X | X | X |  |  |  | X | X | 10 (48) |
| Constipation |  |  |  |  |  | * | X |  | X |  | X | X |  | X | X | X |  | X |  | X |  | 10 (48) |
| Passing gas | * |  | X | X |  |  | X |  |  |  |  | X |  |  |  |  | X | X |  | X | X | 9 (43) |
| Joint pain |  |  |  | * | X |  | X |  |  |  |  |  |  | X |  |  | X |  |  | X |  | 6 (29) |
| Skin rash |  |  |  |  | * |  |  |  |  |  |  | X | X |  |  | X |  | X |  | X |  | 6 (29) |
| Cognitive difficulties/difficulty thinking | * |  |  |  |  | X |  |  |  |  |  | X |  | X |  | X | X |  |  |  |  | 6 (29) |
| Nausea | * |  |  |  |  |  | X |  | X |  |  |  | X |  |  |  | X |  |  |  |  | 5 (24) |
| Sleep disturbance | * |  |  |  | X |  |  |  |  | X |  |  |  |  |  |  |  |  | X | X |  | 5 (24) |
| Other symptoms^a^ |  |  |  | * | * |  | * |  | * |  |  |  |  | * | * |  | * |  |  | * |  | 8 (38) |

*First time symptom reported; X signifies subsequent reports. Concept saturation was achieved after the sixth interview for the most common symptoms. No new symptoms were reported after the twentieth interview.

^a^Other symptoms, reported by 1 or 2 participants each, included arm swelling, black stool, canker sores, dental problems, earaches, feeling overheated, foot dropping, heart palpitations, heartburn/indigestion, lack of appetite, noise in head, numbness/tingling in hands and feet, skin colour changes, vision problems and weight gain.

*CDSD*, Celiac Disease Symptom Diary.

**Supplementary table 3** Development of the original 10-item CDSD (version 1.0)^©^: demographics and clinical characteristics for the cognitive debriefing interview population (*N* = 15)

| **Characteristic** | **Cognitive debriefing interviews**  **(*N* = 15)** |
| --- | --- |
| **Age, years, mean (range)** | 49 (21–80) |
| **Female, %** | 67 |
| **Race/ethnicity, *n* (%)** |  |
| **White** | 13 (86.7) |
| **Hispanic** | 2 (13.3) |
| **Education, *n* (%)** |  |
| **High school/GED** | 2 (13.3) |
| **Trade school** | 0 (0.0) |
| **Some college** | 2 (13.3) |
| **Associate degree** | 2 (13.3) |
| **Bachelor’s degree** | 2 (13.3) |
| **Master’s degree** | 4 (26.7) |
| **Professional/doctorate degree** | 3 (20.0) |
| **Employment status, *n* (%)** |  |
| **Employed full-time** | 9 (60.0) |
| **Employed part-time** | 3 (20.0) |
| **Retired** | 2 (13.3) |
| **Unemployed/seeking work** | 1 (6.7) |
| **Student** | 0 (0.0) |
| **Time since diagnosis, months, mean (range)** | 22 (3–60) |
| **GFD adherence, *n* (%)** |  |
| **All of the time** | 10 (66.7) |
| **Most of the time** | 5 (33.3) |

*CDSD*, Celiac Disease Symptom Diary; *GED*, general equivalency diploma; *GFD*, gluten-free diet.

**Supplementary table 4** Development of the original 10-item CDSD (version 1.0)^©^: demographics and clinical characteristics for the psychometric validation study population (*N* = 202)

| **Characteristic** |  | **Psychometric validation**  **(*N* = 202)**  ***n* (%)** |
| --- | --- | --- |
| **Age category, years**  **18–30** |  | 44 (21.8) |
| **31–40** |  | 42 (20.8) |
| **41–50** |  | 43 (21.3) |
| **51–60** |  | 47 (23.3) |
| **61–70** |  | 13 (6.4) |
| **71–80** |  | 11 (5.4) |
| **81+** |  | 2 (1.0) |
| **Sex**  **Male** |  | 40 (19.8) |
| **Female** |  | 162 (80.2) |
| **Race/ethnicity^a^**  **White** |  | 200 (99.0) |
| **Black/African American** |  | 0 (0.0) |
| **American Indian/Alaska Native** |  | 3 (1.5) |
| **Native Hawaiian/Other Pacific islander** |  | 0 (0.0) |
| **Asian** |  | 1 (0.5) |
| **Hispanic/Latino** |  | 7 (3.5) |
| **Highest level of education**  **Less than high school** |  | 1 (0.5) |
| **High school/GED** |  | 15 (7.4) |
| **Some college (no degree)** |  | 35 (17.3) |
| **Associate degree** |  | 17 (8.4) |
| **Trade school** |  | 2 (1.0) |
| **Bachelor’s degree** |  | 74 (36.6) |
| **Master’s degree** |  | 44 (21.8) |
| **Professional/doctorate degree** |  | 14 (6.9) |
| **Current employment status**  **Employed full-time** |  | 110 (54.5) |
| **Employed part-time** |  | 21 (10.4) |
| **Homemaker** |  | 13 (6.4) |
| **Student** |  | 17 (8.4) |
| **Retired** |  | 21 (10.4) |
| **Unemployed, seeking work** |  | 7 (3.5) |
| **Unable to work owing to illness or disability** |  | 13 (6.4) |
| **Length of illness,^b^ years**  **<1**  **1–10** |  | 52 (25.7)  133 (65.8) |
| **11–20** |  | 13 (6.4) |
| **21–30** |  | 3 (1.5) |
| **31+** |  | 1 (0.5) |
| **Severity of illness^b^**  **Very mild** |  | 12 (5.9) |
| **Mild** |  | 37 (18.3) |
| **Moderate** |  | 81 (40.1) |
| **Severe** |  | 56 (27.7) |
| **Very severe** |  | 16 (7.9) |
| **GFD adherence**  **All of the time** |  | 171 (84.7) |
| **Most of the time** |  | 30 (14.9) |
| **Some of the time** |  | 1 (0.5) |
| **A little of the time** |  | 0 (0.0) |
| **Never** |  | 0 (0.0) |

^a^Respondents could select more than one option for ethnicity. ^b^Length and severity of CeD were self-reported.

*CDSD*, Celiac Disease Symptom Diary; *GED*, general equivalency diploma; *GFD*, gluten-free diet.

**Supplementary table 5** Development of the original 10-item CDSD (version 1.0)^©^: number of participants reporting a symptom at least once in the psychometric validation study (*N* = 202)

| **Symptom** | **Symptom frequency (*N* = 202)**  ***n* (%)** |
| --- | --- |
| **Passing gas** | 128 (63.4) |
| **Abdominal pain** | 125 (61.9) |
| **Fatigue** | 112 (55.5) |
| **Bloating** | 109 (54.0) |
| **Diarrhoea** | 92 (45.5) |
| **Constipation** | 85 (42.1) |
| **Nausea** | 70 (34.7) |
| **Cognitive difficulties/difficulty thinking** | 70 (34.7) |
| **Headache** | 63 (31.2) |
| **Skin rash** | 34 (16.8) |

*CDSD*, Celiac Disease Symptom Diary.

**Supplementary figure 1** Development of the original 10-item CDSD (version 1.0)^©^: symptom occurrence across a 7-day period reported during the psychometric study (*N* = 202)
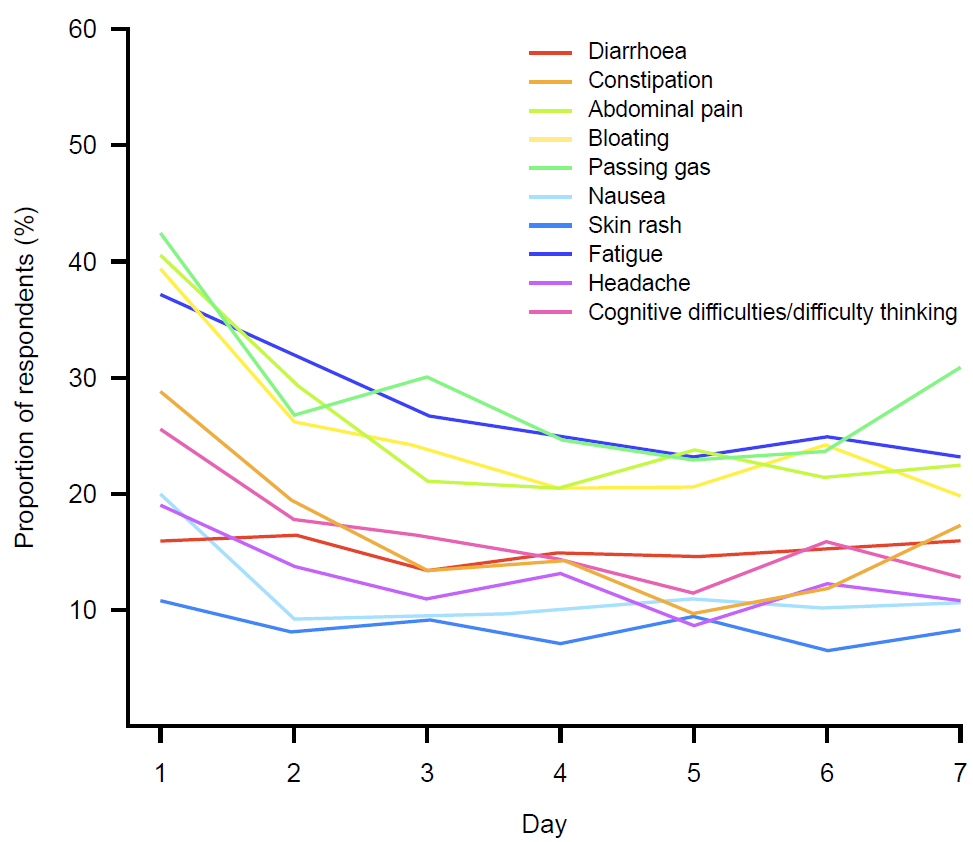


*CDSD*, Celiac Disease Symptom Diary

**Supplementary table 6** Development of the original 10-item CDSD (version 1.0)^©^: demographics and clinical characteristics from the supplemental qualitative interview population (additional concept elicitation, *N* = 10)

| **Characteristic** | **Additional concept elicitation interviews**  **(*N* = 10)** | |
| --- | --- | --- |
| **Age, years, mean (range)** | 49 (27–67) | |
| **Female, %** | 80.0 | |
| **Race/ethnicity, *n* (%)^a^** | |  |
| **White** | 10 (100.0) | |
| **Hispanic** | 2 (20.0) | |
| **Education, *n* (%)** | |  |
| **Some college** | 2 (20.0) | |
| **Bachelor’s degree** | 2 (20.0) | |
| **Master’s degree** | 3 (30.0) | |
| **Professional/doctorate degree** | 3 (30.0) | |
| **Employment status, *n* (%)** | |  |
| **Employed full-time** | 6 (60.0) | |
| **Employed part-time** | 3 (30.0) | |
| **Unemployed/seeking work** | 1 (10.0) | |
| **Student** | 0 (0.0) | |
| **Time since diagnosis, years, mean (range)** | 8.5 (1.3–25) | |

^a^Respondents could select more than one option for ethnicity.

*CDSD*, Celiac Disease Symptom Diary.

**Supplementary table 7** Development of the original 10-item CDSD (version 1.0)^©^: frequency of spontaneously elicited symptom concepts and symptom saturation matrix from the supplemental qualitative interviews (additional concept elicitation, *N* = 10)

| **Participant** | **1** | **2** | **3** | **4** | **5** | **6** | **7** | **8** | **9** | **10** | **Total (%)** |
| --- | --- | --- | --- | --- | --- | --- | --- | --- | --- | --- | --- |
| **Diarrhoea** | * | X | X | X | X | X | X | X | X | X | 10 (100) |
| **Bloating** | * | X |  | X | X | X |  | X | X |  | 7 (70) |
| **Constipation** |  | * |  | X | X | X | X |  | X | X | 7 (70) |
| **Abdominal pain** | * |  |  | X | X | X | X | X |  |  | 6 (60) |
| **Passing gas** | * |  |  |  | X |  |  | X | X |  | 4 (40) |
| **Fatigue** |  | * |  |  | X |  | X |  |  | X | 4 (40) |
| **Skin rash** | * |  |  | X |  |  |  |  | X |  | 3 (30) |
| **Nausea** |  |  |  | * | X |  |  |  |  |  | 2 (20) |
| **Headache** |  | * | X |  |  |  |  |  |  |  | 2 (20) |
| **Sleep disturbance** |  |  |  |  |  |  | * |  | X |  | 2 (20) |
| **Cognitive difficulties/difficulty thinking** |  |  |  |  | * |  |  |  |  |  | 1 (10) |
| **Other symptoms^a^** | * | X |  |  | X | X | X | X | X | X | 8 (80) |

*First time symptom reported; X signifies subsequent reports.

**^a^**Other symptoms, reported by only 1 or 2 participants each, included arm swelling, black stool, canker sores, dental problems, earaches, feeling overheated, foot dropping, heart palpitations, heartburn/indigestion, lack of appetite, noise in head, numbness/tingling in hands and feet, skin colour changes, vision problems and weight gain.


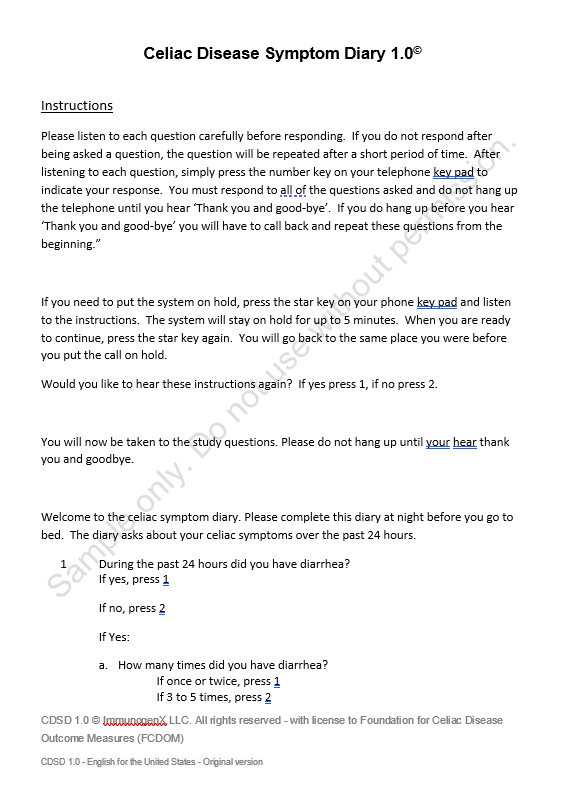


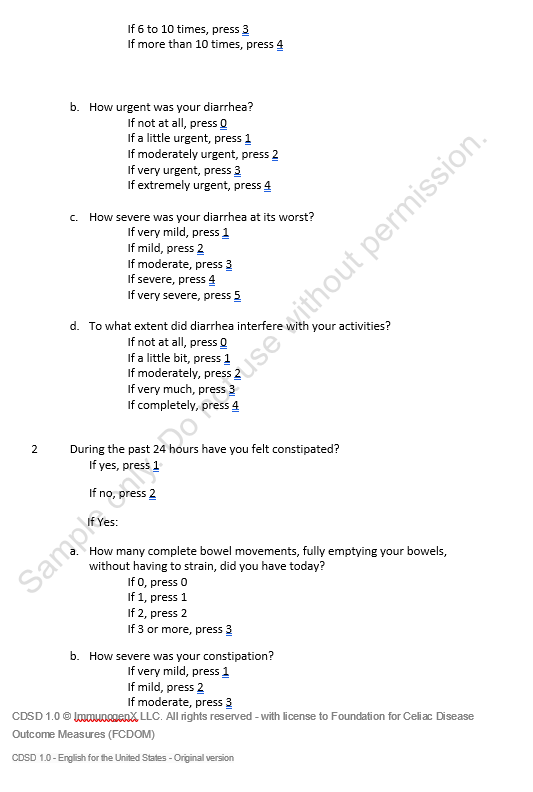


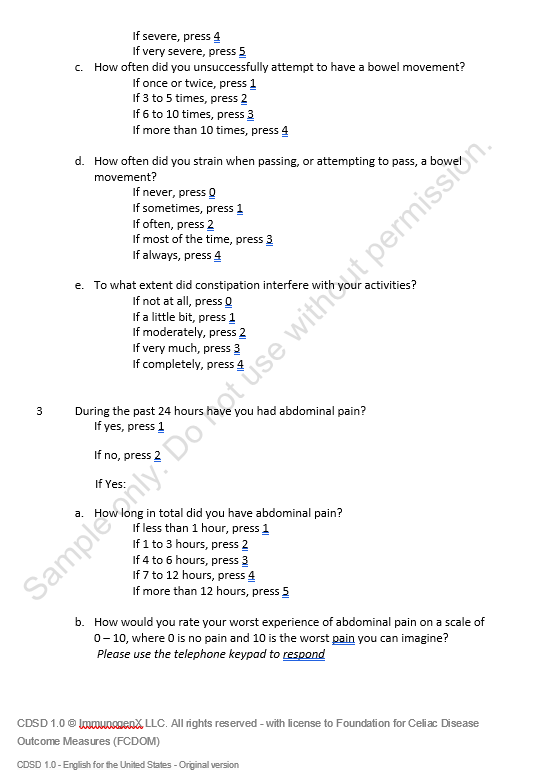


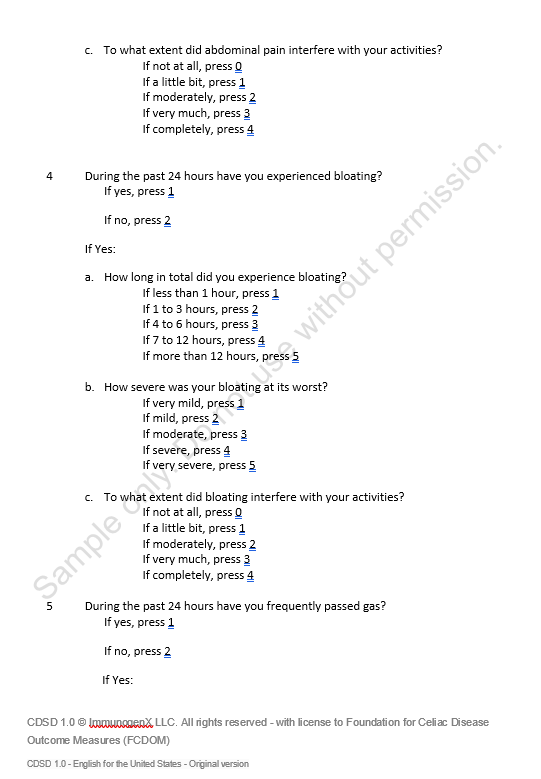


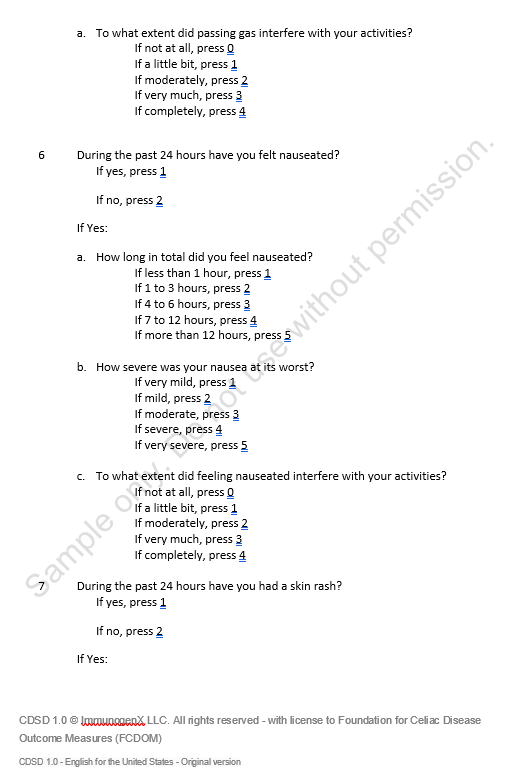


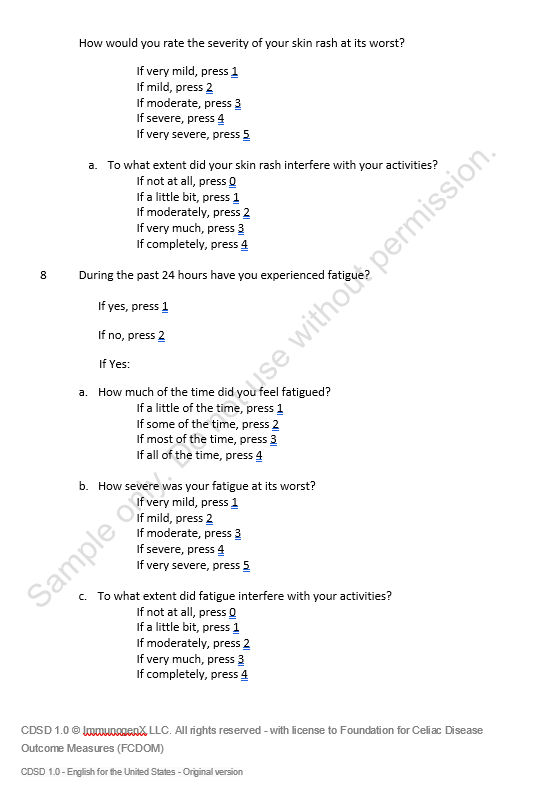


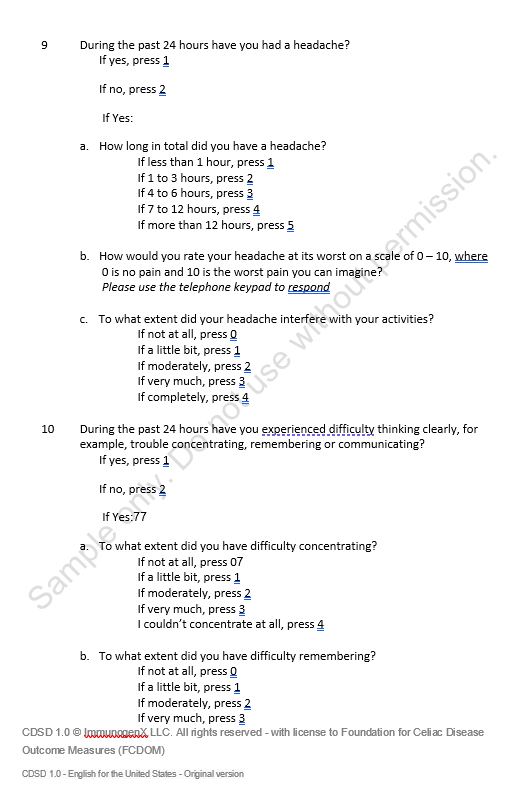


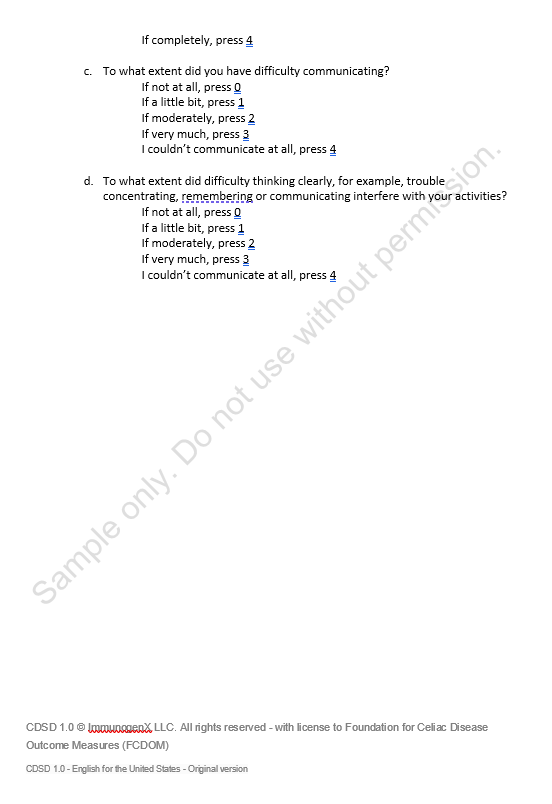


# Abstract 2: Development and validation of CDSD 1.1^©^

Objective:

To modify CDSD 1.0^©^ in response to FDA feedback and conduct additional concept elicitation and cognitive debriefing interviews to further assess content validity and participants understanding of the PRO measure.

Methods:

Non-GI symptom items (cognitive difficulties/difficulty thinking, headache, joint pain and skin rash) and passing gas were removed from CDSD 1.0^©^ in addition to all impact and duration sub-items.

In addition, the word ‘fatigue’ was replaced with ‘tiredness’ along with further clarifications to the wording of the CDSD with regard to spontaneous bowel movements, diarrhoea and bloating. The constipation item was skipped if the participant answered ‘yes’ to experiencing diarrhoea or spontaneous complete bowel movements.

Reference to CeD was removed when asking participants about a specific symptom, so that participants could report on a symptom without having to make an assessment concerning its cause or origin. Finally, the response options were modified so that a score of zero was used to indicate the absence of a symptom.

These changes resulted in a 6-item measure with a recall period of 24 hours. Response options included a numerical scale (0–10) for abdominal pain, a 5-point scale (very mild to very severe) for bloating, nausea and tiredness, and frequency options (0 to ≥10) for diarrhoea and spontaneous bowel movement.

The modified CDSD (CDSD 1.1^©^) was assessed in an additional round of cognitive debriefing interviews in adult participants with CeD (*N* = 15; **Supplementary Table 8**). The inclusion and exclusion criteria were the same as for previous studies.

Results:

Cognitive debriefing interviews (*N* = 15) confirmed that the content of CDSD 1.1^©^ was clear and comprehensible, focused on symptom concepts central to the typical patient’s experience of CeD and that this revised PRO measure could be appropriately interpreted and administered.

FDA feedback (July 2014) on CDSD 1.1^©^ suggested that additional evidence of validity was necessary to use the CDSD to support product labelling of potential CeD treatments for adults and adolescents.

**Supplementary table 8** Development of the 6-item CDSD 1.1^©^: demographics and clinical characteristics from cognitive debrief interviews in participants with CeD (*N* = 15)

| **Characteristic** | **Cognitive debrief interviews**  **(*N* = 15)** |
| --- | --- |
| **Age, years, mean (range)** | 40.1 (21–74) |
| **Female, %** | 86.7 |
| **Race/ethnicity, *n* (%)** | |
| **White/non-Hispanic** | 15 (100) |
| **Hispanic** | 0 (0.0) |
| **Education, *n* (%)** | |
| **High school/GED** | 1 (6.7) |
| **Trade school** | 1 (6.7) |
| **Some college** | 3 (20.0) |
| **Associate degree** | 1 (6.7) |
| **Bachelor’s degree** | 5 (33.3) |
| **Master’s degree** | 4 (26.7) |
| **Professional/doctorate degree** | 0 (0.0) |
| **Employment status, *n* (%)** | |
| **Employed full-time** | 9 (60.0) |
| **Employed part-time** | 0 (0.0) |
| **Retired** | 1 (6.7) |
| **Unemployed/seeking work** | 3 (20.0) |
| **Student** | 2 (12.3) |
| **Severity rating of CeD, *n* (%)** | |
| **Mild** | 3 (20.0) |
| **Moderate** | 5 (33.3) |
| **Severe** | 2 (13.3) |
| **Very severe** | 5 (33.3) |

*CDSD*, Celiac Disease Symptom Diary; *CeD*, celiac disease; *GED*, general equivalency diploma.

Celiac Disease Symptom Diary (CDSD) 1.1^©^


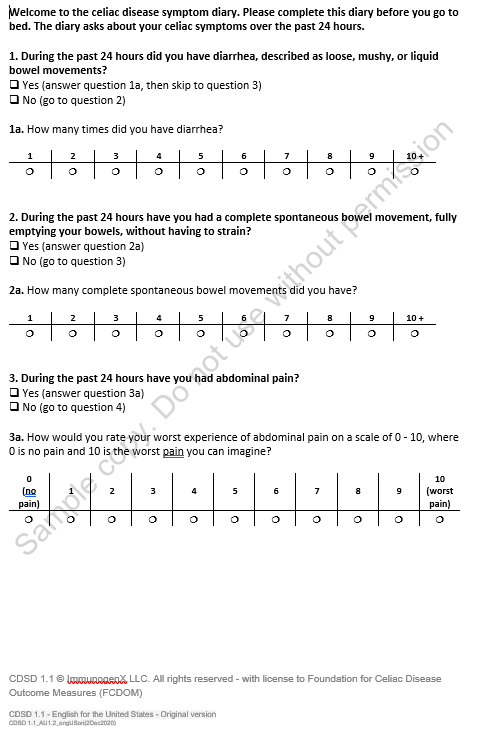


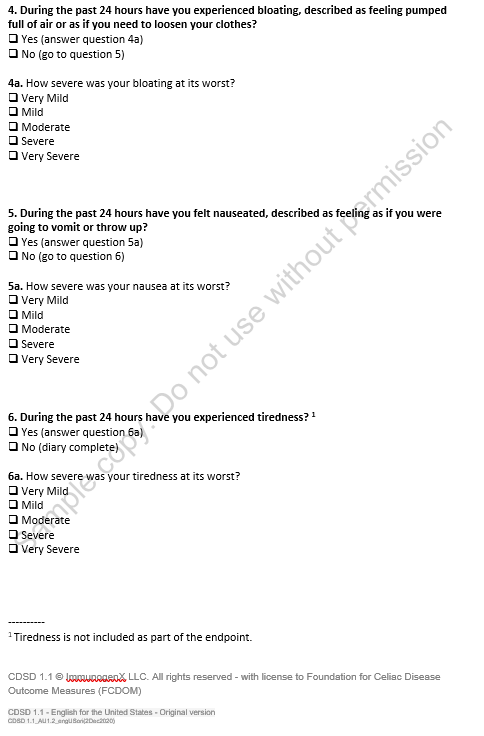


# Abstract 3: Development and validation of CDSD 2.1^©^

Objective:

To further modify and validate the CDSD to reflect FDA and European Medicines Agency (EMA) guidance on the use of PRO measures to support product labelling, including the evaluation of content validity in both adult and adolescent patients with CeD.

Methods:

CDSD 1.1^©^ was further modified to focus on symptom severity scores only. Frequency of bowel movements was removed and a supplementary questionnaire (CDSD 2.1^©^ - Frequency Supplement) was developed to capture information on the frequency of ‘all’ bowel movements and bowel movements classified as ‘Type 6 or 7’ on the Bristol Stool Form Scale (BSFS).^1^ The descriptors of ‘loose, mushy or liquid’ stools were removed. Two item versions for the symptoms of ‘bloating’ and ‘nausea’ were evaluated, one with a further description of each symptom and one without additional descriptions.

The scoring system for all items was modified to 0 (none), 1 (very mild), 2 (mild), 3 (moderate), 4 (severe) and 5 (very severe). The non-GI item ‘tiredness’ was retained, because this was reported at a higher frequency and was considered of higher importance to participants than other non-GI items. Entry of the number of bowel movements was included in the Frequency Supplement (to replace previous options of once/twice/more than three times) as per recommendations from the FDA and EMA.

After the above changes, concept elicitation and cognitive debriefing interviews were conducted in the US with adult (*n* = 16) and adolescent (*n* = 16) participants with CeD (**Supplementary** **Table 9**). The participants were aged ≥18 years (adults) or >12 to ≤17 years (adolescents) with a self-reported clinician’s diagnosis of CeD based on either endoscopy and/or serology, had maintained a GFD for at least 6 months, and reported disease symptoms resulting from inadvertent gluten exposure in the past 3 months. For adolescents, self-reporting of gluten exposure that resulted in symptoms experienced longer than 3 months ago was deemed acceptable if the participants could recall and discuss these experiences.

Three rounds of interviews were conducted, each with a different cohort. During an initial concept elicitation portion of the interview, participants were asked to report on the symptoms that they experienced, as well as identifying symptoms that were most bothersome to them. A cognitive debriefing portion of the interview was then initiated in which participants were asked to review the CDSD in its entirety for content validity (with specific attention to diarrhoea), along with the CDSD 2.1^©^ - Frequency Supplement. Interviews lasted approximately 45 to 60 minutes and were audio recorded and transcribed. Refinements were made between each round of interviews as required.

Results:

Round 1 interviews included 15 participants (adults, *n* = 8; adolescents, *n* = 7), round 2 included 10 participants (adults, *n* = 8; adolescents, *n* = 2) and round 3, conducted via telephone, included 7 participants (adolescents only). Targeted recruitment of younger adolescent participants (to ensure comprehension of items in this age group) was successful, with half of adolescent participants aged 12–14 years (*n* = 8).

Concept elicitation

Most participants (84.4%) stated that they experienced CeD symptoms only upon exposure to gluten, although some individuals (15.6%) stated that they still experienced symptoms without gluten exposure. A similar symptom experience was observed in both adults and adolescents upon exposure to gluten, with abdominal pain (>85%), tiredness (>60%), bloating (≥50%), nausea (≥50%) and diarrhoea (>35%) the most commonly reported symptoms across all participants (**Supplementary** **Table 10**). In adults, diarrhoea was considered the most bothersome symptom (*n* = 4, 27%) followed by abdominal pain and bloating (*n* = 2, 13% each). In adolescents, the most bothersome symptoms were abdominal pain (*n* = 8, 50%) and vomiting (*n* = 4, 25%).

Cognitive debriefing

Feedback was generally consistent between adult and adolescent participants. The most important item reported by adults and adolescents was abdominal pain (**Supplementary Table 11**). Revisions based on participant feedback were made between each round of interviews.

Round 1 in-person cognitive debriefing interviews

Both adult and adolescent participants were easily able to interpret correctly the instructions for completing the CDSD (Instructions: ‘Welcome to the Celiac Disease Symptom Diary. Please complete this diary before you go to bed. The diary asks about your celiac symptoms over the past 24 hours’). A minor modification to further specify ‘each evening’ was added to the instructions for the round 2 interviews.

Seven participants reported that no important symptoms were missing from the CDSD and 3 or fewer participants reported the following symptoms as missing: vomiting (*n* = 3), brain fog (*n* = 2), rashes/bumps (*n* = 2), loss of appetite (*n* = 2) and itching (*n* = 1).

Abdominal pain was reported as the most important item (*n* = 5) and nausea as the least important item (*n* = 4) in the CDSD.

Overall, 14 participants reported no difficulty with the 24-hour recall period. Based on participant feedback, the items that included further descriptions of bloating and nausea were retained for further evaluation in the round 2 interviews. On the basis of feedback from three participants, that vomiting was a key missing symptom, the number of vomiting episodes was added to the supplementary questionnaire (CDSD 2.1^©^ - Frequency Supplement) to be included in the round 2 interviews. In addition, based on the number of participants who reported an inability to distinguish between ‘very mild’ and ‘mild’, the response option ‘very mild’ was omitted for all items in the CDSD for the round 2 interviews.

Constipation was removed as a severity assessment in the CDSD at this stage because it was thought that the question on bowel movement frequency in the CDSD 2.1^©^ - Frequency Supplement better addressed this concept.

Round 2 in-person cognitive debriefing interviews

Participants were able to correctly interpret the instructions. Four participants reported that no important symptoms were missing from the revised CDSD and three or fewer participants reported that the following symptoms were missing: joint pain (*n* = 2), odour of stool (*n* = 1), headaches (*n* = 3), itchiness (*n* = 2) and feeling weak (*n* = 1).

Nausea was reported most frequently as the most important item (*n* = 5) and diarrhoea as the least important (*n* = 3) in the revised CDSD; 4 participants indicated that all items were important.

None of the participants interviewed had problems with the recall period and no additional items were included in the revised CDSD based on feedback received on this round of interviews.

Round 3 telephone cognitive debriefing interviews (adolescents only)

All adolescent participants were able to correctly interpret the instructions (including those aged 12–14 years). Four participants reported that no important symptoms were missing from the revised CDSD and two or fewer participants reported that the following symptoms were missing: headaches (*n* = 2) and ‘a crummy feeling’ (*n* = 1). All participants were able to correctly interpret the term ‘diarrhoea’ and none reported difficulty with the recall period.

Abdominal pain was reported most frequently as the most important item (*n* = 4) and diarrhoea, vomiting, nausea and tiredness as the least important (*n* = 2 each).

On the basis of the feedback received from this round of telephone interviews, some clarifications were made to improve understanding of the questions. Specifically, the word ‘belly’ was added in parentheses after the term ‘abdominal’ in the CDSD and ‘poops’ was added after the term ‘bowel movement’ in the CDSD 2.1^©^ - Frequency Supplement.

Summary

The results of the concept elicitation portion of the interviews provided qualitative evidence that indicates adolescents and adults with CeD report a similar experience with regards to the most common symptoms of the disease. During the cognitive debriefing portion of the interviews, participants of all ages found the CDSD items relevant to their symptom experience, easy to understand and simple to answer. Participants also reported having no difficulty in recalling the symptoms experienced in the past 24 hours. The results of this study provide evidence that the CDSD 2.1^©^ adequately and appropriately assesses the symptoms of CeD in adults and adolescents.

**Supplementary table 9** Development of CDSD 2.1^©^: demographics and clinical characteristics of participants in the concept elicitation and cognitive debriefing interviews (*N*= 32)

| **Characteristic** | **Adults**  **(*n* = 16)** | **Adolescents**  **(*n* = 16)** | **Total**  **(*N* = 32)** |
| --- | --- | --- | --- |
| **Age, years, mean (range)** | 42.5 (19–70) | 14.3 (12–17) | 28.4 (12–70) |
| **Female, %** | 11 (69) | 9 (56) | 20 (63) |
| **Diagnosis method** |  |  |  |
| **Blood test only** | 8 (50) | 9 (56) | 17 (53) |
| **Endoscopy only** | 4 (25) | 3 (19) | 7 (22) |
| **Blood test and endoscopy** | 4 (25) | 4 (25) | 8 (25) |
| **Time since diagnosis, years, mean (range)** | 7.9 (2–22) | 7.9 (0.11–16)^a^ | 7.9 (0.11–22) |
| **Race/ethnicity, *n* (%)** |  |  |  |
| **White** | 12 (75) | 13 (81) | 25 (78) |
| **African American** | 1 (6) | 2 (13) | 3 (9) |
| **Other** | 3 (19) | 1 (6) | 4 (13) |
| **Highest level of education, *n* (%)** |  |  |  |
| **High school** | 1 (6) | NA | 1 (3) |
| **Some college** | 5 (31) | NA | 5 (16) |
| **College degree** | 7 (44) | NA | 7 (22) |
| **Professional degree** | 3 (19) | NA | 3 (9) |
| **Employment, *n* (%)** |  |  |  |
| **Full-time** | 9 (56) | NA | 9 (28) |
| **Part-time** | 3 (19) | NA | 3 (9) |
| **Not employed/retired/ student** | 4 (25) | NA | 4 (13) |

^a^One adolescent was newly diagnosed (6 weeks/0.11 years) owing to difficulties in recruitment.

*CDSD*, Celiac Disease Symptom Diary; *NA*, not applicable.

**Supplementary table 10** Development of CDSD 2.1^©^: symptoms reported by adult and adolescent participants with CeD in concept elicitation interviews (*N* = 32)

| **Symptom** | **Adults**  **(*n* = 16)** | **Adolescents**  **(*n* = 16)** | **Total**  **(*N* = 32)** |
| --- | --- | --- | --- |
| **Abdominal pain (including stomach aches, cramping)** | 14 | 15 | 29 |
| **Tiredness (including fatigue, exhaustion)** | 12 | 10 | 22 |
| **Bloating** | 12 | 8 | 20 |
| **Nausea** | 8 | 12 | 20 |
| **Diarrhoea** | 12 | 6 | 18 |
| **Constipation** | 8 | 5 | 13 |
| **Vomiting^a^** | 6 | 5 | 11 |
| **Rashes^a^** | 6 | 1 | 6 |
| **Headache/migraine** | 4 | 1 | 5 |
| **Passing gas^a^** | 4 | 1 | 5 |
| **Brain fog^a^** | 5 | – | 5 |
| **Inflamed or painful joints^a^** | 3 | – | 3 |
| **Itching^a^** | 3 | – | 3 |
| **Stomach rumblings^a^** | 2 | – | 2 |
| **General malaise/flu-like^a^** | 2 | – | 2 |
| **Irritability^a^** | 2 | – | 2 |
| **Smelly stool^a^** | 2 | – | 2 |

^a^Items not probed upon so all reports spontaneous.

*CDSD*, Celiac Disease Symptom Diary; *CeD*, celiac disease.

**Supplementary table 11** Development of CDSD 2.1^©^: most and least important CeD concepts reported by participants in round 1, 2 and 3 interviews (cumulative results, *N* = 32)

| Item concept | Number of participants who scored item as MOST important | Number of participants who scored item as LEAST important |
| --- | --- | --- |
| Abdominal pain | 9 | – |
| Nausea | 8 | 8 |
| Diarrhoea | 4 | 7 |
| Vomiting | 3 | 2 |
| Episodes of bowel movement | 2 | 1 |
| Constipation | 2 | 2 |
| Bloating | 2 | 5 |
| Tiredness | 2 | 5 |
| Type of bowel movement/stool | 1 | 1 |

*CDSD*, Celiac Disease Symptom Diary; *CeD*, celiac disease.


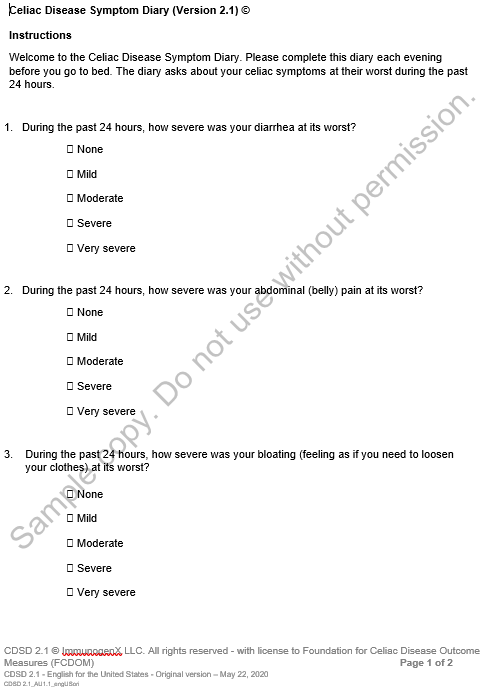


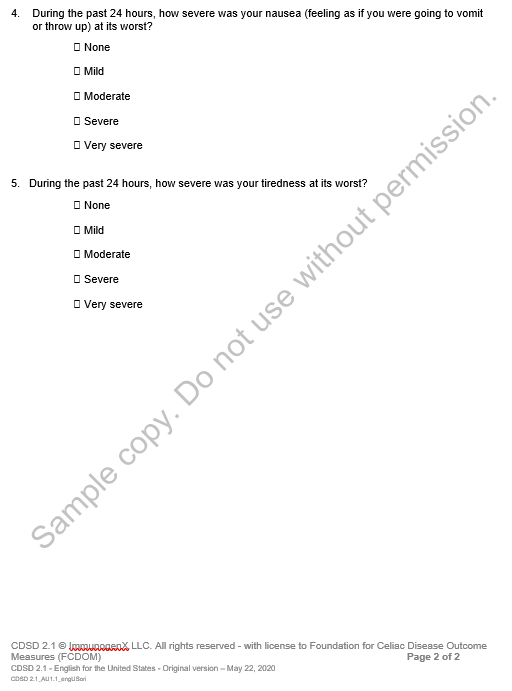


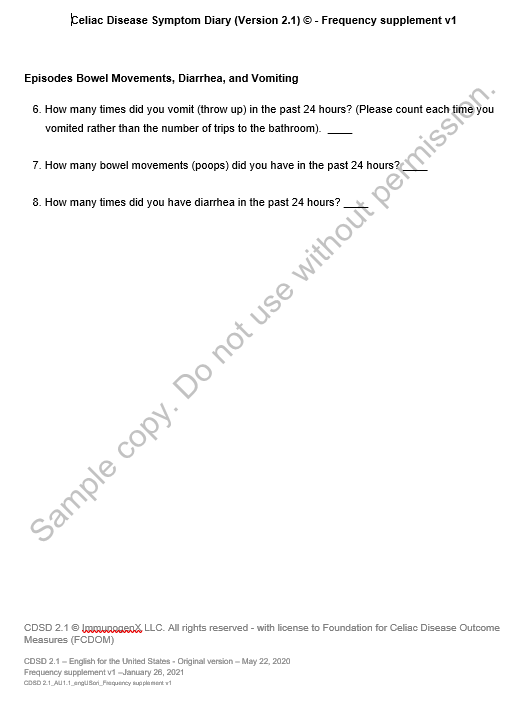


Note: during the interviews undertaken to support development of CDSD 2.1^©^, item 8 of the CDSD 2.1^©^ – Frequency Supplement was worded as ‘bowel movements (poops) that looked like Type 6 or 7’ and relevant pictures from the Bristol Stool Form Scale (BSFS) were provided.

# References

1. Lewis SJ, Heaton KW. Stool form scale as a useful guide to intestinal transit time. *Scand J Gastroenterol* 1997;32:920-4.
